# Characterization of the reproductive strategy of invasive Round Goby (*Neogobius melanostomus*) in the Upper Danube River

**DOI:** 10.1002/ece3.70349

**Published:** 2024-10-01

**Authors:** Melina Klarl, Joachim Pander, Juergen Geist

**Affiliations:** ^1^ Aquatic Systems Biology Unit, TUM School of Life Sciences Technical University of Munich Freising Germany

**Keywords:** gonadosomatic index, invasive species management, reproductive strategy, Round Goby, spawning season, time‐series analysis

## Abstract

Originating from the Black and Caspian seas, the Round Goby (*Neogobius melanostomus*) has become one of the most successful invaders of freshwater ecosystems. In this study, we provide a characterization of the reproductive strategy of an established population of Round Gobies in the Upper Danube river including sex ratio, fluctuations of gonadosomatic index (GSI), analysis of timing of spawning as well as of clutch and egg size. We compare these results to other studies from the native and invaded range. In the Danube, the Round Goby population was found to be female dominated, however fluctuations in magnitude of female bias were observed between months. Monitoring of the population across 1.5 years revealed that GSI was highest from April to June, while lowest values were observed in August and September. Using time‐series analysis, a delayed effect of temperature on GSI was found for females and males, while a quicker response of GSI levels to photoperiod and discharge was observed for females. GSI increased with body size for females and eggs were found to be significantly larger in May, however clutch sizes did not differ between months. Results of a literature review revealed great differences in timing and length of spawning season as well as sex ratio between populations throughout the distribution range, which can probably be explained by climatic and photoperiodic conditions together with the time since invasion and the high plasticity of Round Gobies.

## Graphical abstract

Characterization of the reproductive strategy of round goby (*Neogobius melanostomus*) in the Upper Danube over 1.5 years revealed the interrelations of temperature, discharge and photoperiod with gonadosomatic index and spawning. Differences in sex ratios found between sampling months as well as differences in size‐dependent reproductive investments among sexes can be explained by the nest‐guarding behavior of males. The great plasticity of reproductive and other life history traits across the distribution range of round goby likely explain the invasion success at different environmental conditions.

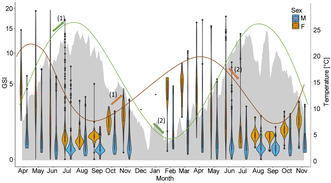

## 1 INTRODUCTION

The spread of invasive species is a major threat to many ecosystems and species diversity around the world, comprising a wide range of species and affected habitats (Keller et al., 2011). Species like the North American Muskrat (*Ondatra zibethicus*), the American Gray Squirrel (*Sciurus carolinensis*), the Chinese Mitten Crab (*Eriocheir sinensis*), the Colorado Beetle (*Leptinotarsa decemlineata*) or the Fall Webworm (*Hyphantria cunea*) are only a few of many species, that have been spread around the world and invaded novel environments (Elton, 2020). The transfer of species is often human‐mediated and the speed and extent of invasions by far exceed natural migration activities (Cerwenka et al., 2021). Aquatic ecosystems are especially at risk as canals and shipping routes are prime corridors for a rapid invasion (Balážová‐L'avrinčíková & Kováč, 2007; Cerwenka et al., 2021).

Six gobiid species, the Round Goby (*Neogobius melanostomus*), the Bighead Goby (*Ponticola kessleri*), the Tubenose Goby (*Proterorhinus marmoratus*), the Racer Goby (*Babka gymnotrachelus*), the Monkey Goby (*Neogobius fluviatilis*) and the Caucasian Dwarf Goby (*Knipowitschia caucasia*) from the Pontocaspian region (Black Sea, Caspian Sea, Sea of Azov) have presumably been spread throughout Europe and North America via ballast water of ships and eggs attached to shipping hulls (Balážová‐L'avrinčíková & Kováč, 2007; Borcherding et al., 2021; Hirsch et al., 2016; Kováč et al., 2009).

Round Gobies are one of the most successful invaders and have colonized many stream and lake ecosystems around the world, e.g. the Great Lakes in North America (Gutowsky & Fox, 2012; MacInnis & Corkum, 2000; Young et al., 2010), the Gulf of Gdansk in Poland (Tomczak & Sapota, 2006), the Baltic Sea (Balážová‐L'avrinčíková & Kováč, 2007; Corkum et al., 2004), the Lower Rhine in Germany (Gertzen et al., 2016), the High Rhine in Switzerland (Kalchhauser et al., 2013), several tributaries of the Lower Danube in Bulgaria (Dashinov & Uzunova, 2021) and the Upper Danube in Germany (Brandner et al., 2018). They are often considered prime model species of aquatic invasion processes (Cerwenka et al., 2023) and their invasion success is attributed to their aggressive behavior, tolerance towards a wide range of environmental conditions regarding salinity, temperature and oxygen levels, a broad diet including their ability to feed on mollusks and their reproductive behavior (Balážová‐L'avrinčíková & Kováč, 2007; Cerwenka et al., 2023; Corkum et al., 2004; Demchenko & Tkachenko, 2017). Round Gobies are batch spawners that spawn multiple times per year and pursue parental care (Kuczyński, 2018; Meunier et al., 2009), which ensures a high survival rate of offspring of up to 90% (Bonisławska et al., 2014). However, population traits like maturation, fecundity, spawning season and the associated invasion success of a population are thought to be highly site‐specific and the status of the population and spread also highly depend on the time since invasion (Brandner et al., 2018; Cerwenka et al., 2023). As prevention strategies are the most effective and economical way of reducing the spread of invasive species (Sepulveda et al., 2011), knowledge of these life‐history traits and site‐specific differences between populations can inform the management of invasive species. As invasions of Round Gobies tend to be a rapid process, only prevention of further introductions can be seen as a promising management approach (Brandner et al., 2018). Even if prevention of initial introduction fails, efforts should be directed to preventing establishment and further spread (Hänfling et al., 2011).

To take appropriate measures against the spread of propagules of invasive species, such as eggs and clutches, information on timing and length of spawning activity is needed. However, such basic knowledge related to reproduction is often missing for invasive species, including the Round Goby in the Upper Danube catchment. Other aspects such as population density, diet and genetics in this system are already well studied (Brandner et al., 2018; Brandner, Cerwenka, et al., 2013; Cerwenka et al., 2017, 2018, 2021), but detailed information on the reproductive strategy, being essential for understanding the development of a population and invasion success, is not yet available. Therefore, the aim of this study was to characterize the reproductive activity and spawning behavior of a Round Goby population in the Upper Danube, where Round Gobies were first detected in 2009 (Brandner, Cerwenka, et al., 2013). We examined reproductive traits like sex ratio, gonadosomatic index (GSI), peaks and length of spawning activity as well as clutch area and size for 1.5 years and compared them to published information from other parts of the native and invaded distribution range. Given that Round Gobies originate from a much warmer climate than the Upper Danube, we hypothesized that they would benefit from increased water temperatures, leading to a rise in their GSI as temperatures rise. Additionally, since female Round Gobies can produce multiple batches of eggs during the spawning season (MacInnis & Corkum, 2000), we predicted that the species would have an extended spawning season from spring to autumn, characterized by multiple peaks in spawning activity. As other goby species have shown a decrease in egg size from early to late in the spawning season (Tamada, 2008), we anticipated that clutches would be larger during peak spawning times. Moreover, we assumed the population would have a balanced sex ratio, consistent with findings from an established population in the Lower Danube (Dashinov & Uzunova, 2021). As we assumed a high level of plasticity for the species, we hypothesized differences in the reproductive strategy between the Upper Danube system and other studies across the distribution range.

## 2 MATERIALS AND METHODS

### 2.1 Study area

**FIGURE 1:**
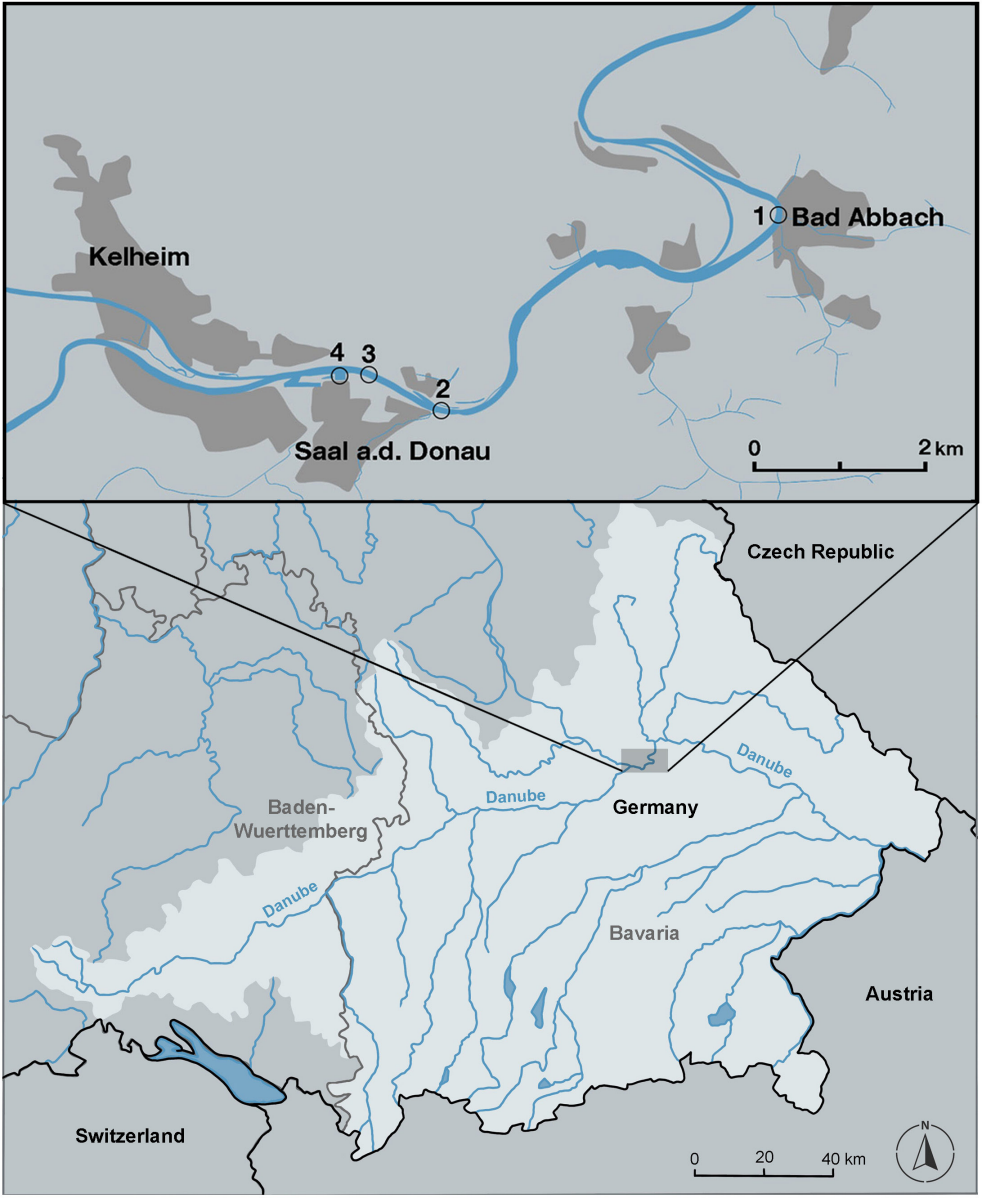
Overview of the Danube catchment area in the southern German states Bavaria and Baden‐Wuerttemberg (light gray). In the detailed map, 1 marks the study site in Bad Abbach, 2 and 3 the study sites in Saal an der Donau, and 4 marks the marina in Saal an der Donau. Electrofishing was conducted at sites 1–3. Spawning traps were placed at locations 1–4.

The Danube is the second longest river with the second largest catchment area in Europe (Flussgebietsgemeinschaft Donau, 2024a). It connects 10 countries and is therefore one of the most important waterways in Europe. The Rhine–Main–Danube Canal connects the catchment of the Danube with that of the Rhine, creating an invasion corridor from the Black Sea to the North Sea. The Danube flows through Bavaria in Germany over a distance of around 400 km, of which around 213 km have been transformed to a federal waterway, which is navigable by large ships (Flussgebietsgemeinschaft Donau, 2024b) (Figure 1). The studied population of Round Goby was identified based on previous population screenings across the Upper Danube river and was located between Bad Abbach (48°56′22.9″ N 12°02′18.3″ E) and Saal an der Donau (48°54′35.9″ N 11°55′27.6″ E) (Figure 1). The sampling site was located in the navigable part of the river, where river banks consisted mainly of artificial rip‐rap structures without canopy cover.

### 2.2 Field sampling

Round Gobies were collected from April 2022 until November 2023 twice a month by electrofishing or baited fish traps. In total, seven electrofishing campaigns between June 2022 and June 2023 were conducted along the rip‐rap structures at the shoreline of the river at sampling locations 1–3 (Figure 1). Three 30 m‐replicate stretches were sampled at each location during electrofishing. On all other sampling dates, baited fish traps were placed along the same stretches in **~**0.4–1.5 m depth for 24 h per sampling date. After collection, all gobies were euthanized using an overdose of the anesthetic MS‐222 (Tricaine), while all native fish were carefully returned to the river. To avoid degradation of tissue, gobies were transported to the laboratory (Aquatic Systems Biology, Technical University of Munich) on ice in a cooler and were frozen afterwards at −20°C until further analysis. On every sampling date, water temperature was measured using a Multimeter (Multi 3430 Set F, WTW Germany). However, as differences between measured temperature and temperature data of the nearby measuring station in Kelheim were ≤0.2°C (Figure 1), temperature of the measuring station was used to get a higher resolution of data (Gewässerkundlicher Dienst Bayern, 2024). Discharge data was obtained from the same measuring station (Hochwassernachrichten Dienst Bayern, 2024).

### 2.3 Sex ratio

For the calculation of sex ratio, only fish from electrofishing were used, as this method is the least selective in terms of fish sex and therefore most suitable for population assessment (Brandner, Pander, et al., 2013). Sex of fish was determined based on the shape of the urogenital papilla, that is broad and blunt in females and longer and pointed with a terminal slit in males (Kornis et al., 2012). Only fish >50 mm were used as sex determination is unreliable for smaller individuals (Brandner et al., 2018; Gertzen et al., 2016).

### 2.4 Gonado‐somatic index (GSI)

For the analysis of the GSI, fish caught by electrofishing and baited traps were used. Like for the calculation of sex ratio, only mature individuals >50 mm were used. A total of 1041 females (size 84 mm ± 21) and 1026 males (size 84 mm ± 21) were dissected. Before dissection, gobies were patted dry with a paper towel, total length was measured to the nearest millimeter and fish were weighed to the nearest 0.001 g using precision scales (Kern ADJ 100‐4). After dissection, the wet weights of the gut content and gonads were recorded. The somatic mass (*M*_s_) and GSI were calculated according to Brandner et al. (2018):

$$M_{s}=M_{t}–\left(M_{\text{gonads}}+M_{g}\right)$$

with *M*_t_ = total body mass and *M*_g_ = gut content mass

$$\mathrm{GSI}=100\times M_{\text{gonads}}\;M_{s}^{-1}$$

### 2.5 Clutch size

**FIGURE 2:**
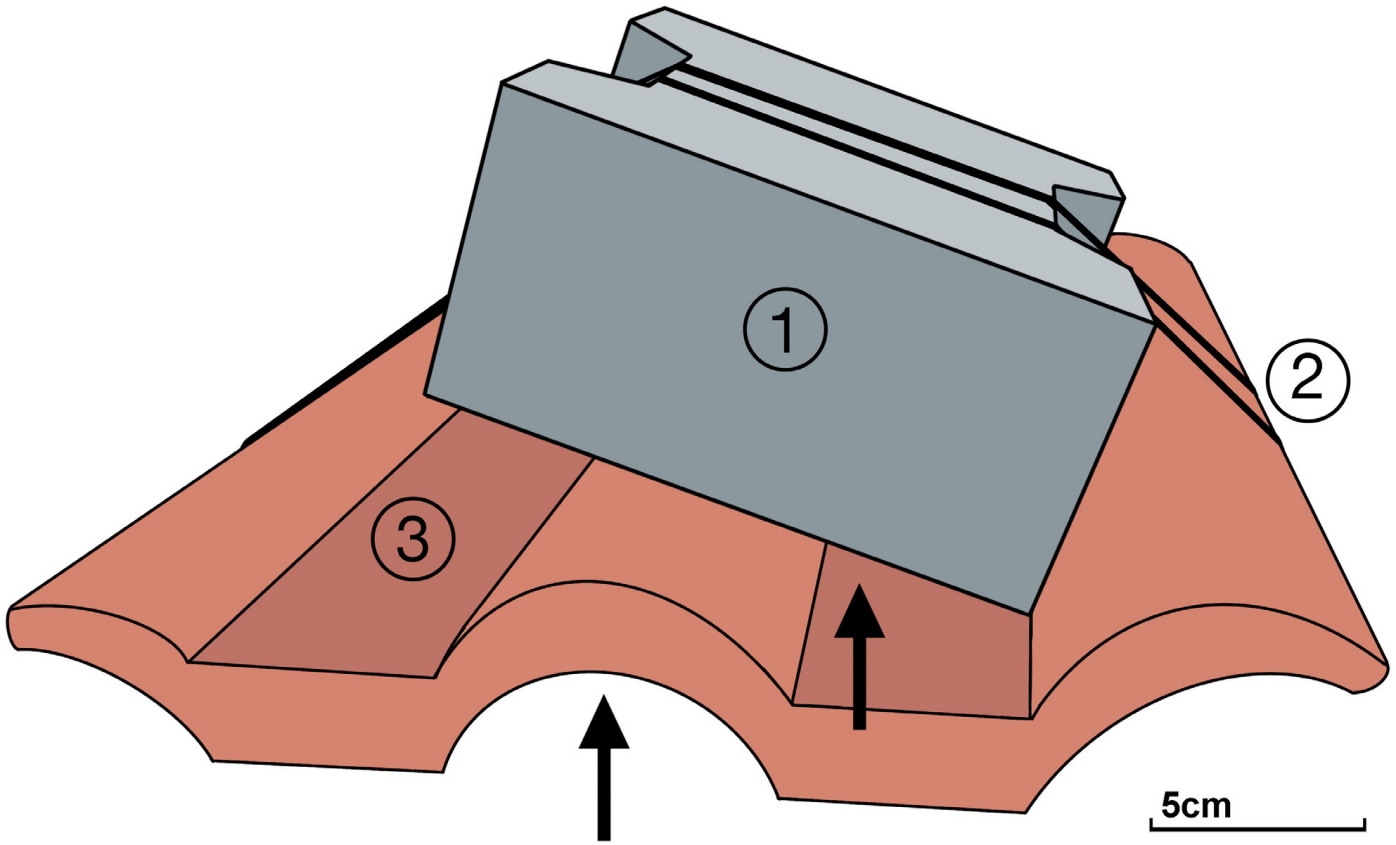
Spawning traps for Round Gobies consisting of a paving stone (1), cable ties (2) and roof tiles (3). Places of egg deposition are indicated by black arrows.

Round Goby eggs were retrieved using spawning traps consisting of a roof tile with a paving stone attached on top with cable ties (Figure 2). This method allowed easy detachment of stones from the roof tiles and access to the clutches without damaging them, as most gobies used the gap in‐between the roof tiles and the paving stones as spawning caves (Figure 2). Ten spawning traps were placed at each location where Round Gobies were collected (sampling locations 1–3, Figure 1) and additionally in the marina in “Saal an der Donau” close to the uppermost stretch of goby collection (sampling location 4, Figure 1). The traps were installed in a depth of **~**0.4–1.5 m in the Danube and ~3 m in the marina, respectively, and were checked for clutches every week. If clutches were present, a translucent foil with an imprinted grid was placed over the clutch and multiple pictures were taken. The photos were used to determine the clutch area in cm^2^ and the average number of eggs per cm^2^. Subsequently, the number of eggs per clutch was calculated (clutch area in cm^2^ × number of eggs per cm^2^). As no different stages of development of eggs were present within the clutches, 10 eggs were randomly taken from each clutch using tweezers and preserved in 96% (v/v) ethanol. Height and width of eggs were measured using a stereo microscope (Binocular Olympus SZX10, Olympus Germany GmbH) with a 20‐fold‐magnitude and the software cellSens (Olympus corporation).

### 2.6 Statistical analysis

#### 2.6.1 Sex ratio

For the analysis of sex ratio, data from the electrofishing campaigns were pooled across sampling sites and analyzed per individual month. Additionally, an overall sex ratio was calculated. To test for differences between the observed sex ratio and an expected 1:1 equilibrium, a chi‐square test was computed.

#### 2.6.2 Gonadosomatic index (GSI) and clutch size

Shapiro–Wilk and Levene test were performed to test for normal distribution and homogeneity of variances of data. In a first step, a regression of GSI with fish size was computed for both female and male Round Gobies to explore the relationship between the two parameters. Further, differences in GSI between fish caught by electrofishing and traps and between sampling sites were examined. As no differences were found (*p* > .05), data was pooled across sampling methods and sites for the analysis of GSI and differences between individual months were analyzed. Since assumptions for parametric tests were not met for GSI, non‐parametric Kruskal–Wallis test with Bonferroni correction for multiple testing was performed. Assumptions of normal distribution and homogeneity of variances were met for clutch and egg size. Therefore, ANOVA with post‐hoc Tukey test was performed to analyze the differences of clutch and egg sizes between sampling dates.

#### 2.6.3 Spawning season—Time‐series analysis

To analyze the influence of water temperature, photoperiod and discharge on GSI of females and males separately, the correlation between these time‐series was examined using cross‐correlation. As data are required to be stationary and prewhitened for cross‐correlation, Kwiatkowski–Philipps–Schmidt–Shin (KPSS) test was used to check for stationarity. All data showed to be stationary, so no further differencing (elimination of a linear trend by computing differences between consecutive observations to make a time series stationary; (Shumway & Stoffer, 2016)) of data was conducted. In this analysis, the influence of one input variable (temperature, photoperiod or discharge) on the output variable (GSI) was tested one at a time. To account for the influence of long‐term trends in the input (temperature, photoperiod or discharge) or the output time‐series (GSI), that may hide or suggest a significant correlation between the two time‐series, prewhitening of data was performed. For prewhitening of data an ARIMA Model (Box & Jenkins, 2013) was fitted to the input time‐series (temperature, photoperiod or discharge), which was then applied to the output time‐series (GSI of females or males) according to Probst et al. (2012) and the cross‐correlation between the two prewhitened time‐series was performed.

As Round Gobies were sampled on 35 occasions within 625 days, one lag in the time‐series analysis (time gap between two time‐series) was calculated as follows:

$$625\;\text{days}/35=17.8\;\left(\approx 18\right)\;\text{days}$$

All analysis were computed using the statistical software R (Ver. 4.1.1, R Core Team, 2024) and significance was accepted for *p* < .05.

### 2.7 Literature review

To evaluate our findings on the Round Goby population in the Upper Danube against other populations, we carried out a comprehensive literature review focusing on the reproductive activity and spawning behavior of Round Gobies across their distribution range. This review utilized the search engines Web of Science and Google Scholar. We developed a set of search strings that included the terms “Round Goby” AND “GSI” OR “GSI” OR “reproduction” OR “spawning behavior” OR “spawning season”. We examined the first 10 pages of search results for pertinent publications and used a snowballing technique to gather additional literature from the references cited in relevant publications. The literature search was conducted between March 3rd and April 30th 2024, with no restrictions placed on the publication date of the literature.

## 3 RESULTS

### 3.1 Fish assemblage in electrofishing campaigns

During seven electrofishing campaigns, a total of 3195 fish representing 33 different species was caught. Seven of these species were non‐native. Gobies represented 55% of the total catch with Round Gobies being the most abundant (45% of total catch). Other goby species were Tubenose Goby (9%), Bighead Goby (0.6%) and Racer Goby (0.09%). The most abundant native species were European Perch (*Perca fluviatilis*), Roach (*Rutilus rutilus*) and Bleak (*Alburnus alburnus*), comprising 27%, 7%, and 2% of the total catch, respectively. More information on fish assemblage is given in Table S1.

### 3.2 Sex ratio

**FIGURE 3:**
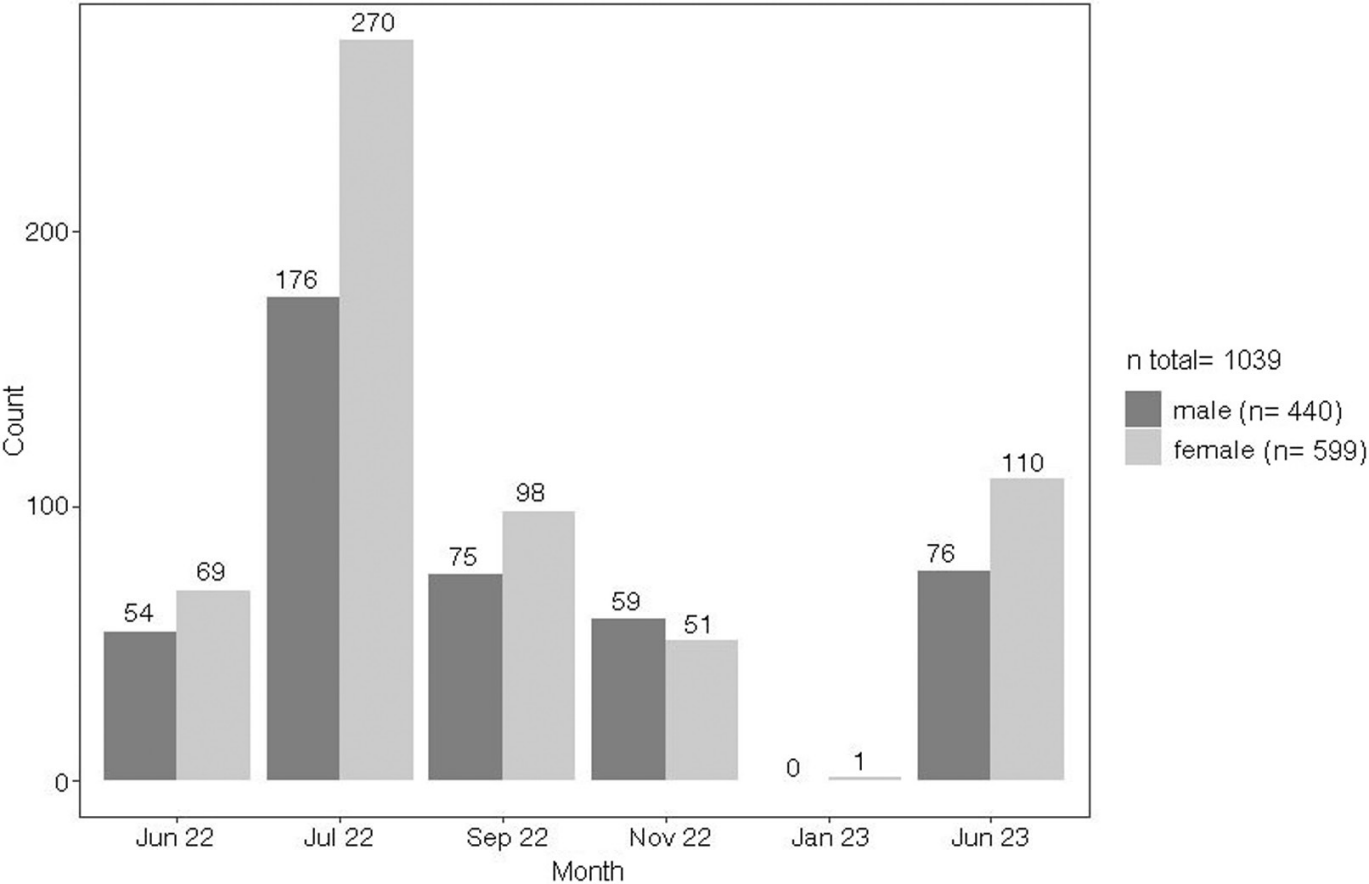
Number of male and female Round Gobies >50 mm caught during 7 electrofishing campaigns. The same effort was expended during all electrofishing campaigns. Two campaigns were conducted in July 2022.

A total of 1039 Round Gobies >50 mm was included in the analysis of sex ratio, whereof 599 individuals were female and 440 were male (Figure 3). This results in an overall female to male sex ratio of 1:0.7. Chi‐square test revealed a significant difference of the observed sex ratio to the expected equilibrium of sexes (*χ* ^2^ = 24.332, df = 1, *p* < .001). Female bias was strongest in July 2022 and June 2023 with a sex ratio of 1:0.65 (*χ* ^2^ = 19.812, df = 1, *p* < .001) and 1:0.69 (*χ* ^2^ = 6.2151, df = 1, *p* < .05), respectively (Table 1; Figure 3). In addition, a slight male bias of 1:1.15 was observed in November 2022, which was not significantly different from equilibrium (*χ* ^2^ = 0.58182, df = 1, *p* > .05) (Table 1; Figure 3). For all other sampling months, sex ratio was not significantly different from equilibrium (*p* > .05).

**TABLE 1:** Sex ratio of Round Gobies >50 mm with minimum (min. GSI), maximum (max. GSI), mean GSI for females and males and mean water temperature (Mean°C) for all sampling month from April 2022 to November 2023.

Month	Sampling method	Sex ratio f:m	Sex of fish	Min. GSI	Max. GSI	Mean GSI ± SD	Mean°C
Apr 22	Traps		Female (*n* = 18)	1.31	14.74	5.47 ± 3.27	10.88
			Male (*n* = 32)	0.11	6.13	0.86 ± 1.29	
May 22	Traps		Female (*n* = 44)	0	19.36	5.44 ± 4.56	17.19
			Male (*n* = 63)	0.04	7.17	1.36 ± 2.04	
Jun 22	Electrofishing and traps	1:0.82	Female (*n* = 92)	0	19.62	3.8 ± 4.68	20.64
			Male (*n* = 81)	0	6.08	1.07 ± 1.81	
Jul 22	Electrofishing (2x)	1:0.65	Female (*n* = 270)	0	17.07	1.26 ± 2.62	22.57
			Male (*n* = 184)	0	7.6	0.4 ± 1.09	
Aug 22	Traps		Female (*n* = 35)	0.08	3.03	0.45 ± 0.51	22.33
			Male (*n* = 40)	0.01	3.42	0.49 ± 0.78	
Sep 22	Electrofishing and traps	1:0.86	Female (*n* = 113)	0	5.01	0.5 ± 0.46	17.18
			Male (*n* = 100)	0	0.92	0.18 ± 0.22	
Oct 22	Traps		Female (*n* = 46)	0.52	2.46	1.3 ± 0.47	13.60
			Male (*n* = 69)	0.02	2.03	0.44 ± 0.51	
Nov 22	Electrofishing and traps	1:1.15	Female (*n* = 81)	0.04	4.42	1.83 ± 0.86	9.66
			Male (*n* = 97)	0	3.15	0.53 ± 0.75	
Dec 22	Traps		Female (*n* = 1)	2.18	2.18	2.18	5.12
			Male (*n* = 0)	—	—	—	
Jan 23	Electrofishing and traps	1:0	Female (*n* = 1)	3.69	3.69	3.69	5.49
			Male (*n* = 0)	0.14	0.14	0.14	
Feb 23	Traps		Female (*n* = 14)	0.06	5.88	3.93 ± 1.8	5.00
			Male (*n* = 27)	0.09	4.78	1.61 ± 1.88	
Mar 23	Traps		Female (*n* = 18)	1.58	8.15	4.85 ± 1.68	7.83
			Male (*n* = 27)	0.02	10.32	1.97 ± 3.01	
Apr 23	Traps		Female (*n* = 52)	0.4	16.47	5.28 ± 3.89	10.13
			Male (*n* = 33)	0.04	17.11	1.84 ± 3.4	
May 23	Traps		Female (*n*=)	0	15.64	4.36 ± 5.09	14.11
			Male (*n*=)	0	5.66	1.08 ± 1.73	
Jun 23	Electrofishing and traps	1:0.69	Female (*n* = 145)	0	17.97	3.31 ± 3.89	20.99
			Male (*n* = 127)	0	8.29	0.81 ± 1.54	
Jul 23	Traps		Female (*n* = 10)	0.11	13.84	2.6 ± 4.78	22.35
			Male (*n* = 28)	0	2.46	0.42 ± 0.6	
Aug 23	Traps		Female (*n* = 14)	0.12	1.56	0.56 ± 0.34	20.34
			Male (*n* = 35)	0	1.66	0.23 ± 0.32	
Sep 23	Traps		Female (*n* = 18)	0.06	0.68	0.44 ± 0.18	18.77
			Male (*n* = 43)	0.01	0.92	0.16 ± 0.17	
Oct 23	Traps		Female (*n* = 53)	0.1	2.01	0.81 ± 0.35	14.39
			Male (*n* = 44)	0.01	1.86	0.51 ± 0.55	
Nov 23	Traps		Female (*n* = 23)	0.11	2.91	1.37 ± 0.65	10.62
			Male (*n* = 18)	0.04	2.63	0.56 ± 0.79	

### 3.3 Clutch size

**FIGURE 4:**
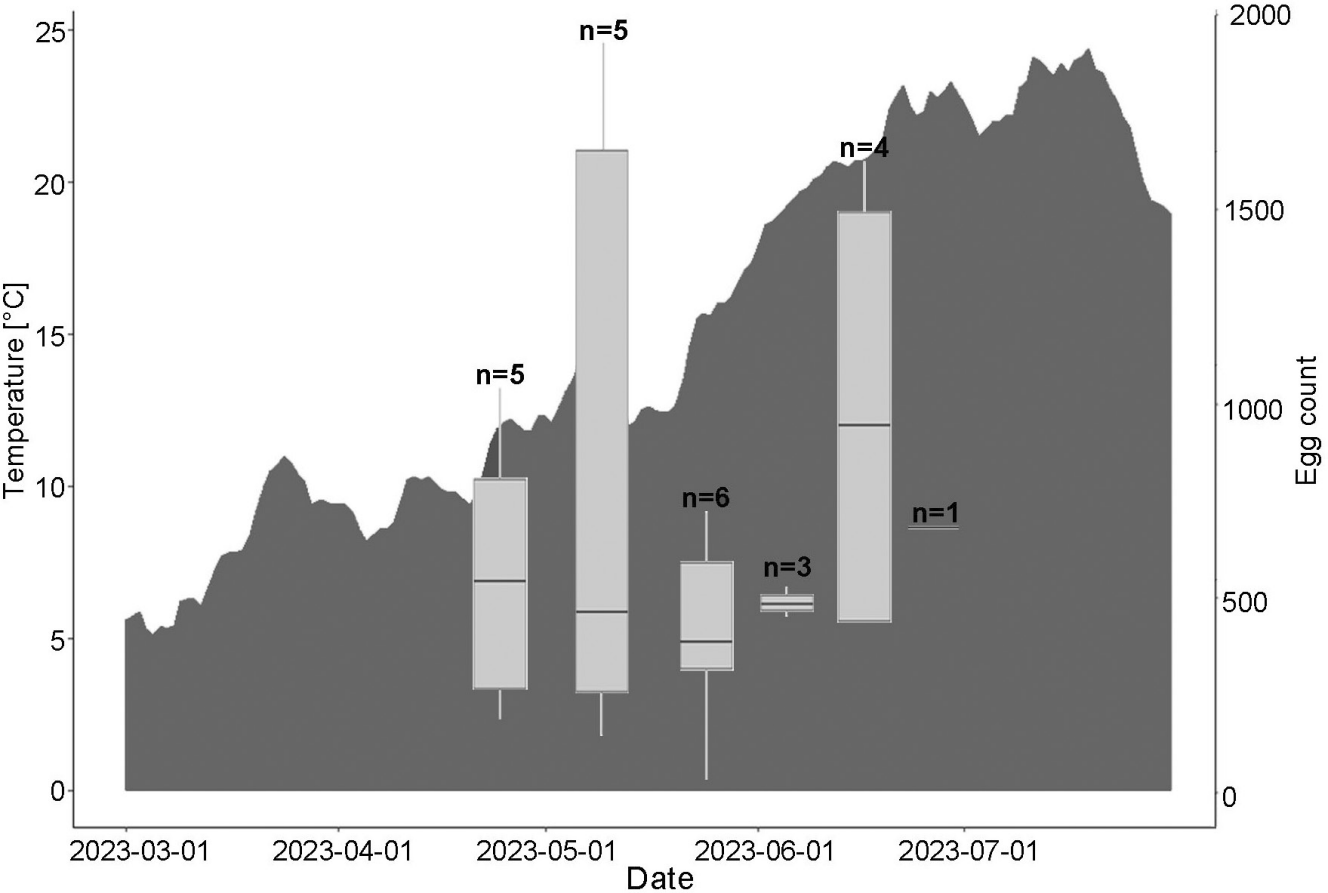
Number of eggs found in clutches in relation to temperature (gray area chart) from March 2023 to July 2023 represented by boxplots. Boxplots show the interquartile range. Median is represented by a horizontal line.

Clutches of eggs were retrieved from the Danube river on six occasions between April and June (Table 2; Figure 4). Before and after that time, no clutches were present in the spawning traps. Between one and six clutches per sampling date were found. Egg numbers per clutch varied widely from 24 to 1918 (Table 2; Figure 4). No differences between clutch sizes (number of eggs) on different sampling dates were found (*p* > .05). Mean size of eggs (height) ranged from 2.74 mm (5th June 2023) to 3.45 (24th May 2023) (Table 2). Differences in the size of eggs (height) were significant for 5th June 2023 and 11th and 24th May 2023, as well as for 26th April 2023 with eggs being significantly larger in April and May (*p* < .05) (Table 2). No statistically significant differences in width of eggs between sampling dates were found (p > .05).

**TABLE 2:** Number of clutches per sampling date with water temperatures in the Upper Danube (Mean temp, °C), mean (*N* eggs mean) and median number of eggs (*N* eggs median), mean and median egg height (mm) and mean and median egg width (mm).

Date	Mean temp (°C)	*N* clutches	*N* eggs mean	*N* eggs median	Egg height mean (mm)	Egg height median (mm)	Egg width mean (mm)	Egg width median (mm)
26‐04‐2023	12.2	5	559.6	533	3.34	3.34	1.71	1.42
11‐05‐2023	13.9	5	881	454	3.34	3.41	1.68	1.70
24‐05‐2023	15.7	6	405	379	3.45	3.43	1.64	1.65
05‐06‐2023	19.2	3	479.7	470	2.74	2.98	1.58	1.52
20‐06‐2023	22.4	4	979.2	936.0	3.03	3.03	1.48	1.46
27‐06‐2023	22.8	1	672	672	2.97	2.98	1.64	1.64

### 3.4 Gonadosomatic index

**FIGURE 5:**
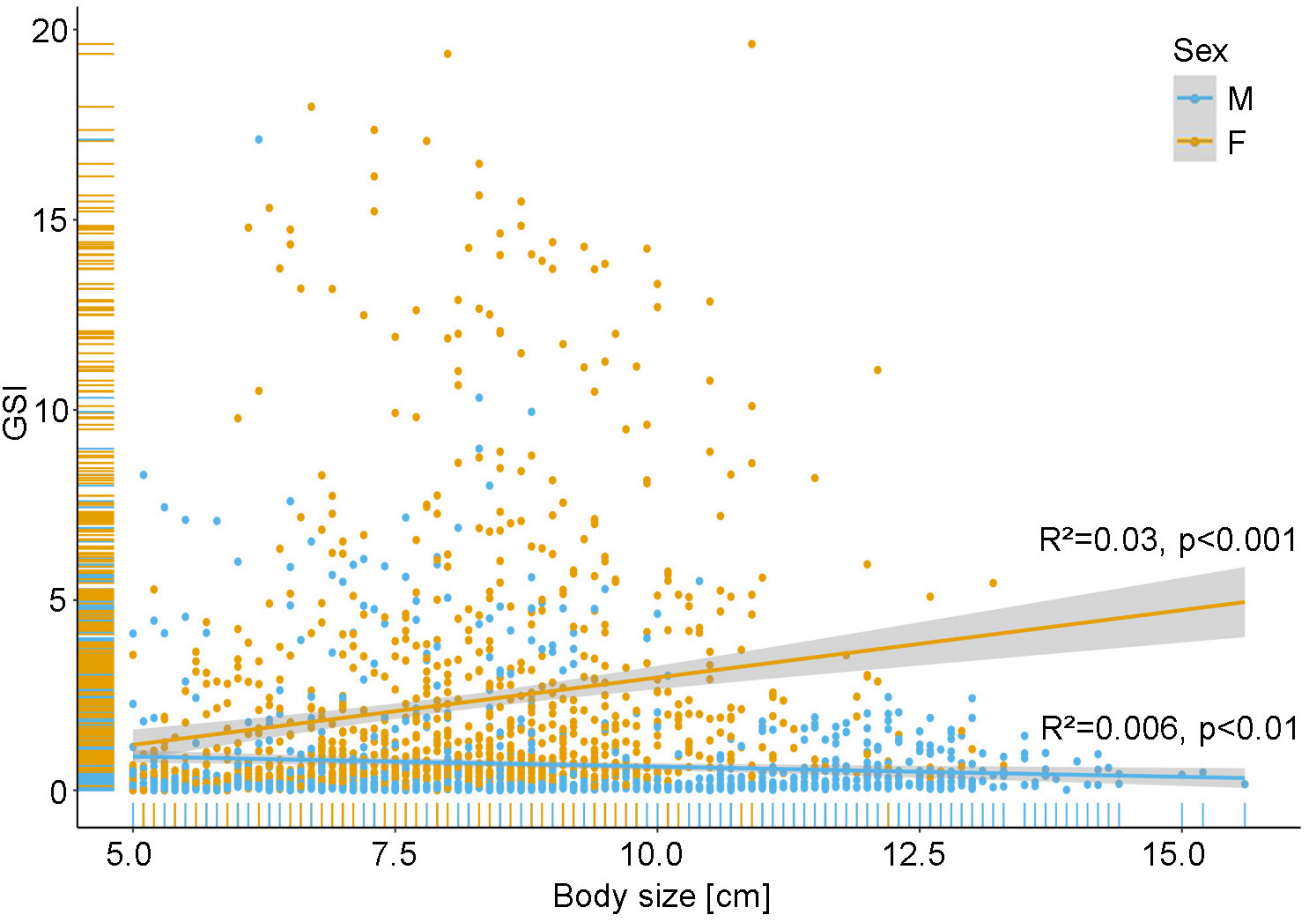
Relationship between body size and GSI for male (blue) and female (orange) Round Gobies. Upper and lower confidence bounds are represented by a gray 95%‐confidence band. No differences in GSI between fish caught by electrofishing or traps were found for fish >5.0 cm (p > .05). Therefore fish caught with both methods were pooled for this analysis.

**FIGURE 6:**
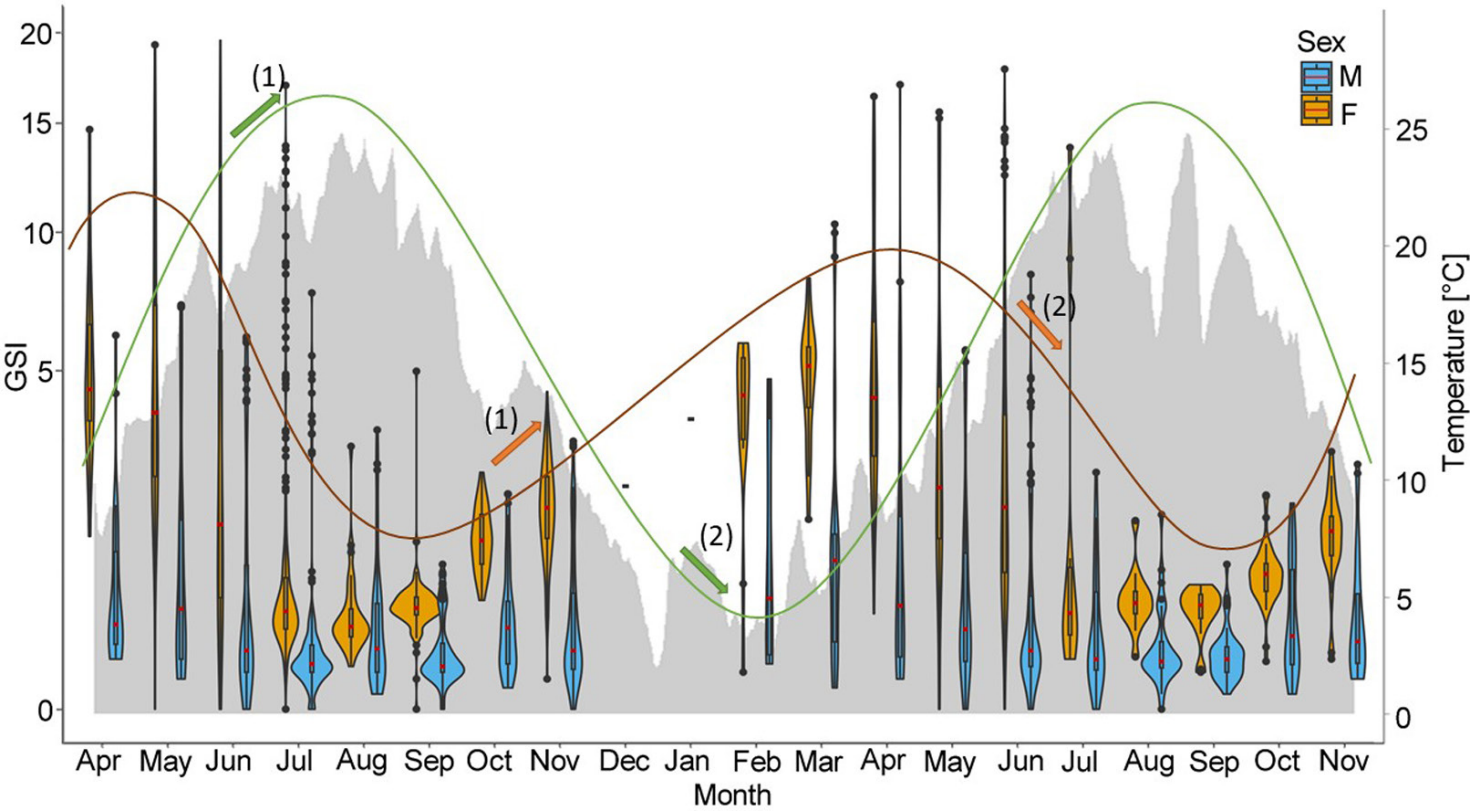
Gonadsomatic Index (GSI) of Round Goby males (blue) and females (orange) in relation to water temperature (gray area chart) from April 2022 to November 2023 represented by violinplots with boxplots. Distribution of data is depicted by violinplots. Boxplots show the interquartile range. Median is represented by a horizontal red line. Outliers are represented by points. The course of the input variable (temperature) is indicated by a green line and the course of the output variable (GSI) is indicated by an orange line. Arrows (1) and (2) show an increase/decrease in temperature and GSI of females and males within 90 days. Arrows marked with the same number belong together.

**FIGURE 7:**
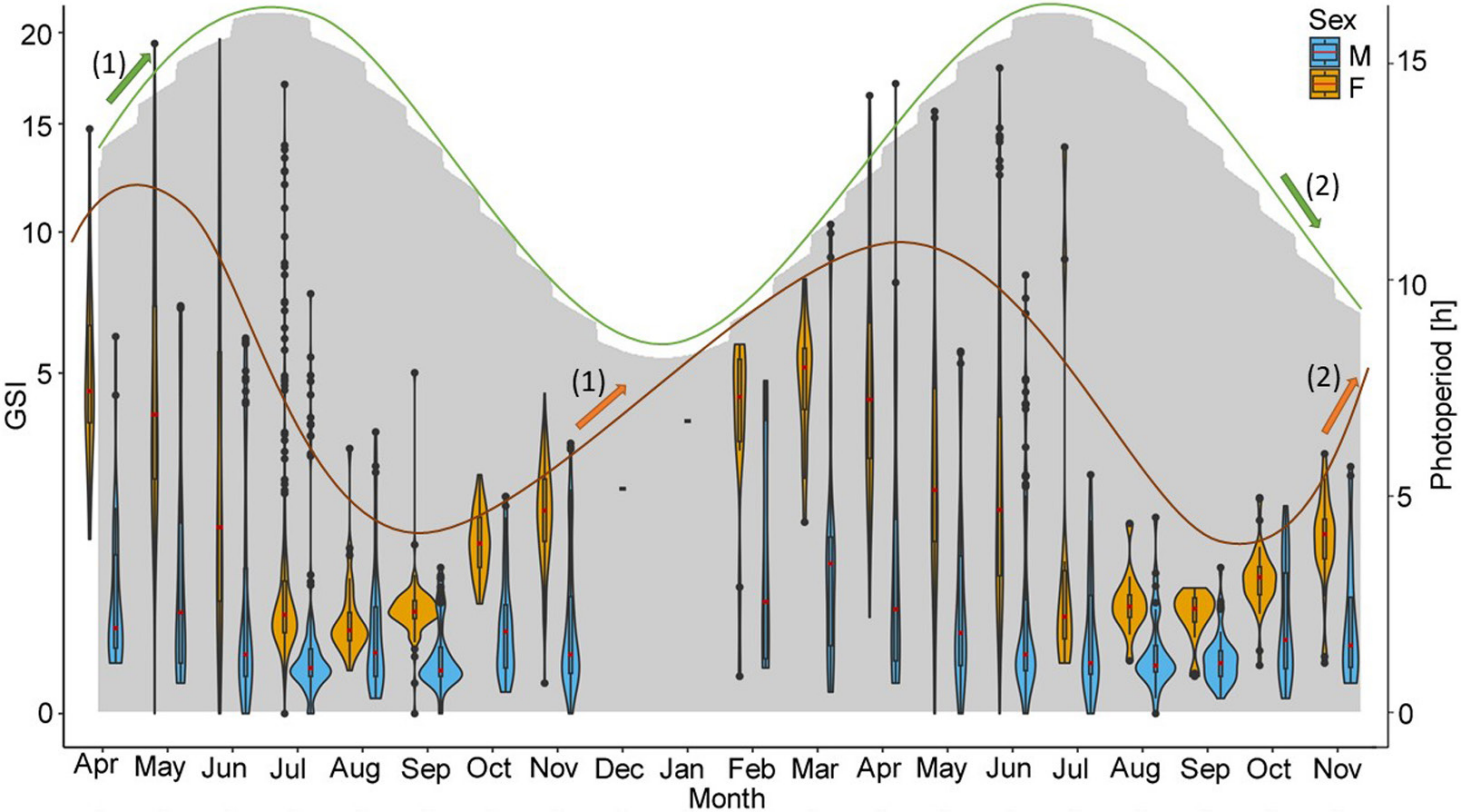
Gonadsomatic Index (GSI) of Round Goby males (blue) and females (orange) in relation to day length (gray area chart) from April 2022 to November 2023 represented by violinplots with boxplots. Distribution of data is depicted by violinplots. Boxplots show the interquartile range. Median is represented by a horizontal red line. Outliers are represented by points. The course of the input variable (photoperiod) is indicated by a green line and the course of the output variable (GSI) is indicated by an orange line. Arrows (1) show an increase in photoperiod and GSI of females with a time lag of 198 days. Arrows (2) show a decrease in photoperiod and an increase in GSI of females within 0–18 days. Arrows marked with the same number belong together.

**FIGURE 8:**
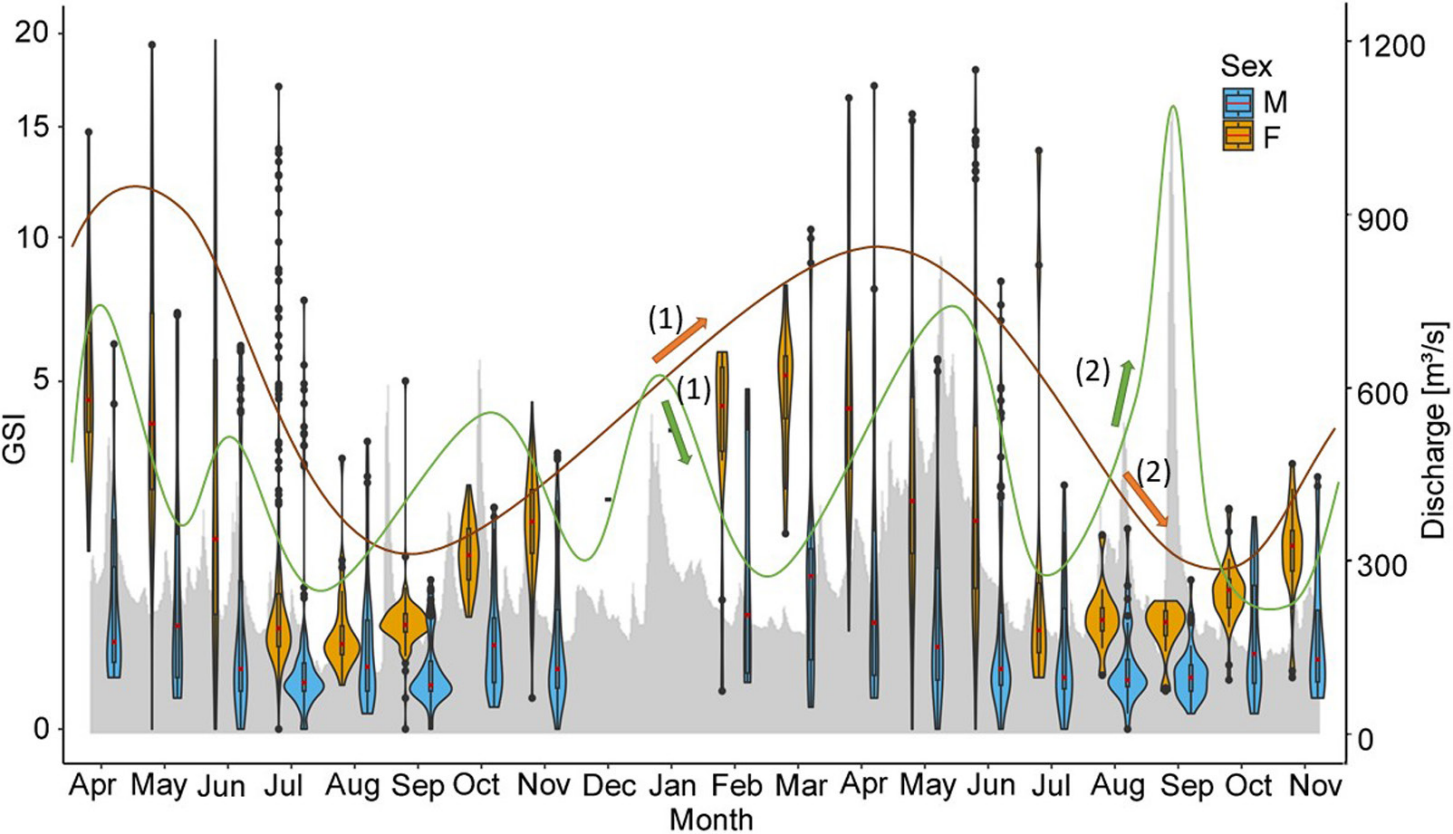
Gonadsomatic Index (GSI) of Round Goby males (blue) and females (orange) in relation to discharge (gray area chart) from April 2022 to November 2023 represented by violinplots with boxplots. Distribution of data is depicted by violinplots. Boxplots show the interquartile range. Median is represented by a horizontal red line. Outliers are represented by points. The course of the input variable (discharge) is indicated by a green line and the course of the output variable (GSI) is indicated by an orange line. Arrows (1) and (2) show an increase in discharge and a decrease in GSI of females within less than 18 days. Arrows marked with the same number belong together.

For female Round Gobies, a significant positive relationship between the body size and GSI was found (*R* ^2^ = .03, *p* < .001) (Figure 5). In contrast, a negative relationship was found in Round Goby males, which was also significant (*R* ^2^ = .006, *p* < .01) (Figure 5). In 2022, a peak in the overall GSI (for males and females) was observed in May at a mean water temperature of 17.2°C, a photoperiod between 14:39 h and 15:56 h and discharge between 212 and 389 m^3^/s (Figures 6, 7, 8). GSI was also high in April and June, indicating a peak during spring (Table 1; Figures 6, 7, 8). Lowest values were found during summertime in August and September when water temperatures ranged from 17.2 to 22.3°C and photoperiod decreased from 15:09 h to 11:46 h (Table 1; Figures 6 and 7). At that time, discharge ranged from 106 to 605 m^3^/s, whereby the highest discharge represents only a short peak (Figure 8). In 2022, no differences were observed between April, May and June, but significant differences were found between April and July (*p* < .05) and May and July (*p* < .001). Towards the end of the year, GSI started to increase again and another peak was observed in April 2023 at a mean water temperature of 10.1°C, a photoperiod between 12:55 h to 14:35 h and a rather high discharge ranging from 251 to 632 m^3^/s (Table 1; Figures 6, 7, 8). Lowest values were again observed in August and September at mean water temperatures of 18.8–20.3°C, suggesting a consistent pattern across years (Table 1; Figures 6, 7, 8). In 2023, significant differences were found between April and all month from June–November (*p* < .05).

For female Round Gobies, GSI levels ranged from 0.01% to 19.62%. While highest mean GSI levels were observed in April of both years, lowest levels were found in August 2022 and September 2023 (Table 1; Figures 6, 7, 8). Differences in GSI levels between highest and lowest values were significant for both years (*p* < .05).

For male Round Gobies, GSI levels between 0.01% and 17.11% were observed. Highest levels were evident in May 2022 and March 2023, while lowest GSI was observed in September of both years (Table 1; Figures 6, 7, 8). Differences between highest and lowest levels were significant for both years (*p* < .05). Overall, GSI was always higher for females than for males (*p* < .05) (Table 1; Figures 6, 7, 8).

### 3.5 Spawning season—time‐series analysis

Significant positive cross‐correlation for water temperature and GSI was found for females and males. The highest influence of temperature on GSI was found at a lag of 5 for both sexes (90 days, CCF for females = 0.38, CCF for males = 0.59) (Figure 6). In contrast, a significant negative cross‐correlation was found for photoperiod and GSI of females at a lag of 0 (less than 18 days, CCF = −0.45) and 1 (18 days, CCF = −0.42). A positive cross‐correlation was found at a lag of 11 (198 days, CCF = 0.53) (Figure 7). For males, neither a significant positive nor negative cross‐correlation was found for photoperiod and GSI. For discharge and GSI of females, a significant negative cross‐correlation was found at a lag of 0 (less than 18 days, CCF = ‐0.48), while no correlation was found for discharge and GSI of males (Figure 8). For male Round Gobies, temperature clearly had the greatest influence on gonad development, whereas for females, the strongest influence was found to be photoperiod, followed by discharge and temperature.

### 3.6 Literature review

Aside from our study, we identified 12 studies that examined the spawning behavior of Round Gobies in detail. The gathered studies included information about the sampling of Round Gobies, such as the sampling location, invasion status (native or invasive), timing and method of sampling, and bottom substrate, as well as reproductive activity data like spawning period, peaks in reproductive activity, GSI, fecundity and sex ratio (Table 3). While most of the studies (*n* = 10) examined the populations of Round Gobies in their invasive range, only two studies from their native range were found (Aydin, 2021; Macun, 2017). These two populations were found to be male‐dominated, while most invasive populations were female‐dominated (Table 3). Additionally, only a single peak in reproductive activity was observed in the native range. In contrast, multiple peaks were recorded in locations like the Gulf of Gdańsk (Tomczak & Sapota, 2006), the Upper Detroit river (MacInnis & Corkum, 2000) and the tributaries of the Lower Danube (Dashinov & Uzunova, 2021) (Table 3). Although similar mean GSI levels for female and male Round Gobies were found between the native and invasive range (e.g., tributaries of the Lower Danube, Bulgaria, and Lake Karaboğaz, Turkey), the onset and duration of the spawning season varied not only between native and invasive ranges but also within the native and invasive range (Table 3).

**TABLE 3:** Comparison of reproductive traits of Round Gobies in different parts across the native and invaded distribution range: location of sampling, invasion status, sampling time and method used for Round Goby collection, bottom substrate on which Round Gobies were caught, spawning period and peaks in reproduction (spawning pattern). Mean GSI is given for females and males, as well as fecundity of Round Goby females and overall sex ratio of the sampled populations. Cited studies are categorized into studies on invasive and native populations and grouped by location, where possible.

Location	Status	Sampling time	Sampling method	Bottom substrate	Spawning period	Spawning pattern	GSI (mean)	Overall sex ratio (f:m)	Literature
Invasive populations									
Upper Danube, Germany	Invasive; established	April 2022–November 2023	Electrofishing and minnow traps	Rip‐rap structures	April–June	Single peak	**Females** 0.44 ± 0.18–5.47 ± 3.27 **Males** 0.14 ± 0–1.97 ± 3.01	1.43:1	This study
Upper Danube, Germany	Invasive; established and invasion front	2010–2015; August – September	Electrofishing	Rip‐rap structures	N/A	N/A	**Females** established area: 0.72 ± 0.32; invasion front: 0.67 ± 0.18–0.81 ± 0.28 **Males** established area: 0.26 ± 0.23; invasion front: 0.23 ± 0.27–0.35 ± 0.27	Established area: 1.14:1; invasion front: 0.83:1–1.79:1	Brandner et al. (2018)
Upper Danube, Germany	Invasive; established and invasion front	2009–2011; March–June and August–October	Electrofishing	Rip‐rap structures	N/A	N/A	**Females** established: 4.3 ± 5.0; invasion front: 2.0 ± 3.3–3.6 ± 4.1 **Males** established area: 0.3 ± 0.8; invasion front: 0.1 ± 0.1–0.7 ± 1.7	Established area: 1.11:1; invasion front: 1.30:1–1.43:1	Brandner, Cerwenka, et al. (2013)
Side‐arm of the middle Danube, Slovakia	Invasive, established	October 2008–July 2010	Angling	Gravel and silt; rip‐rap structures	April– Mid‐July	Single peak	**Females** 0.31–21.51 **Males** N/A	N/A	Hôrková and Kováč (2015b)
Iskar, Yantra and Vit rivers, Bulgaria (tributaries of the Lower Danube)	Invasive; invasion front	March 2017–May2018	Electrofishing	Single boulders, cobble, pebbles and sand	March–June	Multiple peaks	**Females** 1.88 ± 1.39–8.22 ± 2.66 **Males** N/A	1.37:1	Dashinov and Uzunova (2021)
Lower Rhine, Germany	Invasive; established	2011–2013; April–October	Beach seining net	Sand and gravel	Mid March – Mid September	Single peak	**Females** maximum: 5.3 ± 4.8 **Males** N/A	N/A	Gertzen et al. (2016)
Kiel Canal, Germany	Invasive; established	May–October 2011; April to October 2012	Beach seining and demersal trawl	Sand and mud	May‐end of June	Single peak	**Females** maximum: 8.13 ± 5.44 **Males** 1.46 ± 2.23	1.3:1	Hempel et al. (2019)
Gulf of Gdańsk, Poland	Invasive; established	July–October 1999 and March–August 2000	Fyke‐nets	N/A	April–July	Multiple peaks	**Females** 0.2–32 **Males** 0.8–11	0.6:1	Tomczak and Sapota (2006)
Otonabee river, Canada and Moselle river, France	Invasive; established and invasion front	Otonabee: May and July 2008 and 2011 Moselle: March–July 2014	Angling and beach seining	Sand, gravel and rocks	Otonabee: May–July Moselle: March–July	Single peak	**Otonabee** **Females** established: 0.01–0.08 invasion front: 0.06–1.2 **Males** N/A **Moselle** **Females** established: 0.05–0.12 invasion front: 0.06–0.1 **Males** N/A	N/A	Masson et al. (2018)
Trent river, Ontario, Canada	Invasive; established and invasion front	2007–2008; June–August	Angling and beach seining	Small and large rocks	June–August	Established: single peak Invasion front: multiple peaks	**Females** established: 1.99 ± 0.35 (SE) invasion front 2.80 ± 0.35 (SE) **Males** N/A	N/A	Gutowsky and Fox (2012)
Upper Detroit river, USA	Invasive, established	May–October 1996	Otter trawl, angling	Cobble and Sand	Before sampling started‐July	Multiple peaks	**Females** 1.50–8.90 **Males** N/A	N/A	MacInnis and Corkum (2000)
Native Populations									
Lake Karaboğaz, Turkey	Native	August 2011–August 2012	Trammel nets	N/A	April‐early September	Single peak	**Female:** mean: 8.25 maximum in April: 15.0 **Males** mean: 1.12 maximum in May: 3.3	0.62:1	Macun (2017)
Southern Black Sea, Ordu province, Turkey	Native	July 2017–June 2018	Trammel net	N/A	March–May	Single peak	**Females** mean: 1.19 ± 1.22; maximum in April: 3.898 **Males** mean: N/A maximum in April: ~1.2	0.56:1	Aydin (2021)

## 4 DISCUSSION

In this study, the reproductive strategy of an established invasive Round Goby population in the Upper Danube river was characterized and the literature review revealed pronounced differences to other populations and an overall great level of plasticity in this species. Traits like sex ratio, clutch size, the GSI and time and length of spawning season (Table 2; Table 3) together with the predominant fish assemblage (Table S1) were determined for the Upper Danube region representing an important invaded area for this and other invasive species (Cerwenka et al., 2023). As evident from the comparison of data from the Upper Danube with other parts of the native and invaded distribution range, differences in environmental conditions such as climate, photoperiod and discharge, can have a strong influence on the reproductive strategy of a species and consequently the ecology and evolution of the Round Goby. These differences and the associated high plasticity of Round Gobies in their life history traits, e.g. in sex ratio, demography, feeding strategy and parasitic load not only vary with region, but also along invasion gradients within single systems (Brandner, Cerwenka, et al., 2013; Gutowsky & Fox, 2011; Masson et al., 2018).

### 4.1 Fish assemblage

Round Gobies were by far the most abundant species in the sampled area in this study, followed by Tubenose Gobies and a small percentage of Bighead and Racer Gobies. While Racer Gobies (*Babka gymnotrachelus*) were first recorded in the Upper Danube in 2011 (Haertl et al., 2012), they were not caught in previous studies in the Upper Danube (Brandner et al., 2018; Brandner, Cerwenka, et al., 2013). Brandner et al., 2018 found Barbel (*Barbus barbus*) and Chub (*Squalius cephalus*) to be the most abundant native species in the Upper Danube. However, as large predators like European Perch (*Perca fluviatilis*) and Pike‐perch (*Sander lucioperca*) are able to use Round Gobies as new food source, their abundances often increase following a goby invasion (Cerwenka et al., 2023; Young et al., 2010), which is in line with the findings in this study where European Perch was the most abundant native species.

### 4.2 Sex ratio

In this dataset of an established population of Round Gobies from the Upper Danube river, an overall female biased sex ratio of 1:0.7 (f:m) was found. When the invasion front was first discovered in our study area in 2009, already a similar sex ratio of 1:0.77 was found (Brandner, Cerwenka, et al., 2013). Comparison of sex ratios revealed that populations in the native range were male‐dominated, while most invasive established populations were female‐dominated (Table 3). Several studies reported a strong female bias of Round Goby Populations in the Upper Danube (Brandner, Cerwenka, et al., 2013), the Kiel Canal and the Upper Elbe (Hempel et al., 2019; Janáč et al., 2019). In contrast, a male bias was found for established populations in Poland (Tomczak & Sapota, 2006), the United States (Corkum et al., 2004; Young et al., 2010) as well as for the native range of Round Gobies (Aydin, 2021; Macun, 2017). Since environmental factors such as temperature are primary drivers of sex determination in fish (Geffroy & Wedekind, 2020), variations in environmental conditions across different distribution areas may account for these differences in sex ratios. The differences between studies can further be explained by different sampling methods used, the differences in substrate composition in the sampling area and time of sampling. While electrofishing is known to be the least selective method for sampling, other methods like angling and minnow traps are more selective with a lower catch per unit effort (Brandner, Pander, et al., 2013). Furthermore, substrate type was found to have an influence on the abundance of reproductive Round Goby males, with boulder and sand being preferred over cobble (Young et al., 2010). In our study, sampling was conducted along the shoreline of the Upper Danube, where mostly rip‐rap structures were present, which Round Gobies prefer as spawning substrate. Since we used the least selective sampling method to catch the fish used for the calculation of sex ratio, a bias in our results due to the sampling method used is unlikely. However, an influence of the substrate is conceivable. As evident from our dataset, monthly fluctuations of the observed sex ratio in Round Goby populations are possible, which can be explained by the nest‐guarding of males and other biological features. Especially during spawning season when males guard the nests, they are less mobile and can remain hidden in their nests when getting stunned from electrofishing (Dashinov & Uzunova, 2021; Hempel et al., 2019). This aligns with the findings for the studied population in the Upper Danube. While the overall sex ratio was female biased, analysis of individual sampling month revealed a more balanced sex ratio. Fluctuations in sex ratio were also reported for a male dominated population, where male bias was less pronounced in spring and sex ratio was equal in later summer (Tomczak & Sapota, 2006). Also Hempel et al. (2019) found the highest percentage of females in June, while sex ratio was more balanced in September and October.

Studies comparing invasion fronts with established areas further revealed that sex bias is most pronounced at the invasion front (Brandner, Cerwenka, et al., 2013; Gutowsky & Fox, 2011), while established population were often found to be more balanced in sex ratio (Brandner et al., 2018; Brandner, Cerwenka, et al., 2013; Gutowsky & Fox, 2011; Hempel et al., 2019). The overall sex ratio in our study was rather balanced, even though we found a more or less pronounced sex bias between months. Stronger dispersal tendencies of one sex or another, which leads to a sex‐bias at the front of an invasion, can evolve when the spatio‐temporal variability in competition becomes different for males and females (Gros et al., 2009). Therefore, time since invasion seems to play a critical role in determining sex ratios and is especially important, as female‐biased populations may experience different growth dynamics compared to those with a male‐biased sex ratio. Female‐biased populations are not constrained by the availability of females for reproduction, while a strong male‐biased sex ratio can result in reduced offspring production due to the limited number of females, potentially leading to a sharp decline in population size (Brown et al., 2015). Therefore, analyzing the sex ratio of a population can provide insights into its growth dynamics.

However, most of the studies on reproductive traits of Round Gobies only collected specimens at specific time points (Table 3), mostly in spring and summer (Brandner et al., 2018; Cerwenka et al., 2018; Masson et al., 2018; Tomczak & Sapota, 2006). In contrast, our dataset with additional sampling points beyond the spawning season for the Upper Danube clearly suggests that the time of sampling requires continuous (or at least high‐resolution) data for a representative assessment. Given the great level of variability in spawning times identified in this study, it is hardly possible to get an objective result for the overall sex ratio of a population when only short‐term studies are conducted. Therefore, results should always be interpreted with regards to timing and length of sampling periods.

### 4.3 Clutch size

High GSI of Round Gobies in the Upper Danube was observed at water temperatures ranging from 4.9 to 17.2°C, while clutches were found at temperatures between 12.2°C (April 2023) and 22.8°C (June 2023). Compared to findings from the Lower Rhine, where Round Gobies were reported to spawn at water temperatures between 6.9 and 21.9°C (Gertzen et al., 2016), the onset of spawning in the Upper Danube appeared at a considerably higher temperature. However, the onset of spawning varied widely between years in the Lower Rhine and no dependency of spawning onset with water temperature was found there (Gertzen et al., 2016). Further, clutch sizes in our study ranged from very small (24 eggs), possibly due to interruption of spawning, to large with up to 1918 eggs, probably deposited by multiple females. While some studies found females producing six to seven smaller batches of eggs with an average of 163 oocytes (Dashinov & Uzunova, 2021) and smaller nests of 683 eggs of three females (Meunier et al., 2009), others reported a high number of 300–5000 eggs per female (Marsden & Jude, 1995). Since the number of eggs produced per female does not vary with season (MacInnis & Corkum, 2000), this could explain why no differences in the number of eggs per clutch were detected between sampling dates in our study. Also, due to the small sample size and high variation in this study, identifying patterns in the number of eggs per clutch would be challenging and all results should be taken carefully. However, egg sizes were found to vary with season. The largest eggs were present in May (mean height: 3.45 mm), which is at the upper limit of egg sizes (mean: 1.78‐4 mm) reported by other studies (Bonisławska et al., 2014; Marsden & Jude, 1995). Besides different stages of embryonic development influencing height and width of Round Goby eggs (Bonisławska et al., 2014), a correlation for female size with egg size, as was found for other goby species (Mazzoldi et al., 2002), is also likely for the Round Goby. The larger egg size observed in May may be attributed to larger females spawning earlier, which could account for the differences in egg size between the sampling dates in this study. The large size of Round Goby eggs, that was found in this and other studies allows Round Gobies to already resemble adults at hatching and skip a proper larval stage, which can enhance their chances of survival (Bonisławska et al., 2014; MacInnis & Corkum, 2000) and may partly explain their invasion success in many places.

### 4.4 Gonadosomatic index

Highest GSI in the Upper Danube was found in May 2022 and April 2023 in this study, similar to observations in the Lower Danube, Bulgaria, where peaks in the GSI were observed in February and May (Dashinov & Uzunova, 2021) and in the Trent‐Severn Waterway, Canada, where the highest values were found after winter (Houston et al., 2014). In the native range of gobies, spawning season was also observed between March and May with a maximum GSI in April and a minimum reached in August (Aydin, 2021). Similar to the native range and to findings in the Kiel Canal and the Lower Danube, where the lowest GSI was reported in September (Hempel et al., 2019) and from August to November (Dashinov & Uzunova, 2021), the lowest GSI levels in this study were found in August and September, when water temperatures peaked. The observed progressive decrease in GSI between the maximum and minimum levels instead of a rapid decline is typical for multiple spawning species (Rinchard & Kestemont, 1996) and was also observed in Hamilton Harbor, where the proportion of reproductive gobies decreased between May and September, before slightly increasing again in October for Round Goby males (Young et al., 2010).

While GSI was positively related to total length of females, a negative trend was found for males. This can possibly be explained by females not only producing more but also larger eggs with increasing body size. For the Marbled Goby (*Pomatischistus marmoratus*) for example, not only a positive correlation for female size with number of released eggs, but also for female size with egg size was found (Mazzoldi et al., 2002). The same could apply to Round Gobies and explain the increase in GSI with increasing female body size. The slight decrease in male GSI with body size could be caused by an allocation of energy resources towards somatic growth once a certain threshold of sperm production is reached. This would lead to a slight decrease in GSI with increasing body size. Since larger males are able to guard larger nests, as was found for the Marbled Goby (Mazzoldi et al., 2002), a larger body size is likely more advantageous for males than resource allocation to additional sperm production.

In this study, maximum GSI levels of up to 19.62 and 17.11 were found for female and male Round Gobies, respectively. GSI levels are usually higher in non‐native populations compared to native ones (Ondráčková et al., 2010). In the native range, a maximum GSI of 15.0 was found for females, while males showed a maximum GSI of 3.3 (Macun, 2017). Since the invaded ranges of gobies are often highly modified water bodies like the Upper Danube river in this study, they likely benefit from the prevailing environmental conditions. It is well known that anthropogenic habitat alterations can favor the invasion success of non‐native species and erase the advantage of local environmental adaptation of native species (Byers, 2002). As Round Gobies experience less interspecific competition compared to their native range, this can explain a higher energy allocation towards reproduction and a higher GSI in the invaded range as was found in our study. The invasion front was discovered in our sampling area in 2009 and a mean GSI of 2.0 ± 3.3 and 0.1 ± 0.1 was reported for females and males, respectively (Brandner, Cerwenka, et al., 2013). GSI levels are highly influenced by the time since invasion, with GSI being higher at the invasion front (Brandner et al., 2018; Gutowsky & Fox, 2012). However, it must be noted that the invasion front was discovered in autumn 2009 and therefore also the gobies used for analysis were sampled in autumn, when GSI tends to be lowest. Compared to our study, GSI calculated for September, October and November was lower for females in both sampling years than reported by Brandner, Cerwenka, et al. (2013), though for males GSI was higher in autumn of both years. Apart from time since invasion, also environmental perturbations (Hôrková & Kováč, 2015a, 2015b), prey availability and population density can influence GSI (Gutowsky & Fox, 2012) and can explain higher levels of GSI even after a longer time since invasion. Even long established populations of invasive species can experience a rapid population decline after a rapid population growth in a more or less regular boom‐and bust‐cycle (Simberloff & Gibbons, 2004; Strayer et al., 2017), which could be the case for Round Goby populations. Reduced intraspecific competition then allows higher energy allocation towards lipid storage in autumn and superior allocation towards reproduction in spring (Houston et al., 2014) These factors demonstrate how environmental conditions can have a significant impact on the reproductive biology of Round Gobies by influencing the allocation of energy towards reproduction. They can possibly also explain fluctuating GSI levels along an invasion gradient instead of a continous decrease in GSI.

### 4.5 Spawning season and time‐series analysis

In this study, Round Gobies were found to spawn from April to June. Climatic conditions are likely a determining factor for onset and length of the spawning period. While for example, populations in the Middle Danube and in the Kiel Kanal also spawned for short periods of time from April to June (Hempel et al., 2019; Hôrková & Kováč,  2015b), Round Gobies in the Moselle river and the Lower Rhine showed an extended spawning period from March to July and even September (Gertzen et al., 2016; Masson et al., 2018). Surprisingly, even two native populations in Turkey exhibited rather different spawning periods from each other, from March to May in the southern Black Sea (Aydin, 2021) and from April to early September in a brackish coastal lake connected to the Black Sea (Macun, 2017). Further, a study by Masson et al., 2018 showed that, while two different Round Goby populations in North America and Europe exhibited similarities regarding life history traits such as reproductive investment, the onset of the spawning period of the examined populations still happened 2 months apart from each other. Therefore, even if climatic conditions do not influence the general life history of Round Goby populations, an influence on timing and length of spawning appears evident.

In our study, an increase in GSI was seen after a rise in water temperature within 90 days for female and male Round Gobies, which was likely the result of the progressing maturation of gonads (Tomczak & Sapota, 2006). While some studies described temperature as the most important factor in promoting spawning behavior (Kuczyński, 2018) and found an increase in spawning activity with increasing temperature (Pennuto et al., 2010; Tomczak & Sapota, 2006), others found the spawning season to be shorter at warmer temperatures (Gertzen et al., 2016). The varying impact of water temperature on spawning activity may be attributed to the specific conditions to which Round Gobies are adapted. Research on Round Goby populations in the North American Great Lakes Basin showed that their sensitivity to increased temperatures can differ based on their environmental adaptations (Reid & Ricciardi, 2022).

Further, in contrast to other studies, where no influence of photoperiod on spawning activity was reported (Gertzen et al., 2016), we found a slow increase of GSI with increasing length of photoperiod within 198 days and a rapid decline in GSI within 0–18 days for female Round Gobies, which probably marked the end of spawning activity. As photoperiod and water temperature are correlated with each other, it is difficult to separate the influences of these two factors on gonadal development and spawning behavior. The interplay between temperature and photoperiod frequently serves as a cue for ideal spawning conditions in fish. While these factors typically act in concert, there are instances where temperature can exert a dominant influence, potentially overriding the effects of photoperiod on reproductive timing and behavior. We examined the influence of both factors on GSI separately, which however might lead to an overestimation of the influence of one or the other. Overall, temperature appears to be one of the most crucial factors in promoting maturation of gonads, while photoperiod seems to trigger the onset of spawning. Apart from heterogeneity in climate and photoperiod, other factors such as male behavior to avoid niche overlaps may also play a role in explaining the observed differences in onset of spawning activity between studies (Gertzen et al., 2016).

In our study, we further found a significant negative influence of high discharge on GSI of females. GSI levels of females dropped rapidly after an event of high discharge, while no influence on GSI of males was found. Especially in spring and autumn 2023, high water discharge occurred in our study area and it is likely that the timing of such perturbations strongly influences the reaction of Round Gobies. Hôrková and Kováč (2015b) showed that female Round Gobies increase their reproductive effort within the same year or demonstrate enhanced reproductive effort in the subsequent year depending on whether rising water levels and increased flow velocity appear during or after the spawning season. High water discharge can alter environmental conditions in water bodies, potentially causing stress in fish. During increased discharge, the energy demand for other vital processes, such as maintaining position in the water, rises and female fish are often more affected by these changes than males. As our results show, female Round Gobies invest proportionally more energy into production of eggs with increasing body size, while sperm production and therefore the energy demand for reproduction decreases with increasing body size (Figure 5). Consequently, female fish may need to divert energy from gonadal development to ensure these basic functions. This redistribution of energy resources can lead to a more pronounced decline in GSI in females compared to males.

Beyond that, we found a single peak in spawning activity for the Round Goby population in the Upper Danube and also several other studies reported a single peak in spawning activity (Table 3). Three studies observed multiple peaks in established populations (Dashinov & Uzunova, 2021; MacInnis & Corkum, 2000; Tomczak & Sapota, 2006), while one found multiple peaks at the front of an invasion and a single peak in the established part of the population (Gutowsky & Fox, 2012). These differences can likely not only be explained by time since invasion, but also by environmental perturbations such as sudden temperature drops or rising water levels and flow velocities, which can result in the production of an extra batch of eggs or increased number of oocytes (Hôrková & Kováč, 2015b).

Given the variability between Round Goby populations in different distribution areas, which can probably be attributed for by differences in climatic conditions, photoperiod and occurrence of environmental perturbations, it is especially important to consider those factors trying to predict a species response to environmental changes in regards to a changing climate.

## 5 CONCLUSIONS

The results of this study show the great reproductive plasticity of Round Gobies on a population level. In contrast to other parts of the distribution range, the spawning season of Round Gobies in the Upper Danube appears to be rather short with the highest GSI levels in April and May, which indicates a single peak in spawning activity in spring. While climatic and photoperiodic conditions seem to play an important role in the onset and duration of the spawning season of Round Gobies, perturbations like high discharge can determine reproductive effort and spawning patterns, which can explain the differences across the current distribution range. Not only differences in timing and length of spawning season as well as in sex ratio were evident, but also the spawning patterns with single or multiple peaks in spawning activity differed between populations. Basic knowledge on the reproductive strategy of Round Gobies as shown for the Upper Danube can inform managers and contribute to development of effective measures to stop the further spread of Round Goby eggs by means of different distribution vectors, such as ships and ballast water. In light of climate change, the reproductive behavior of Round Gobies is likely to undergo changes. In some regions, shifting climates could lead to a redistribution of Round Gobies due to unfavorable conditions, while in other areas, conditions may become more conducive to their reproduction. Whether they benefit or are disadvantaged by rising temperatures may depend on their current distribution and the conditions to which they are adapted.

## AUTHOR CONTRIBUTIONS

**Melina Klarl:** Conceptualization (equal); data curation (lead); formal analysis (lead); funding acquisition (equal); methodology (equal); visualization (lead); writing – original draft (lead). **Joachim Pander:** Conceptualization (equal); methodology (equal); validation (equal); writing – review and editing (equal). **Juergen Geist:** Conceptualization (equal); funding acquisition (equal); methodology (equal); resources (equal); supervision (lead); writing – review and editing (equal).

## FUNDING INFORMATION

This research was funded by Deutsche Bundesstiftung Umwelt (DBU) (Grant number: 20021/735).

## CONFLICT OF INTEREST STATEMENT

The authors declare that there are no conflicts of interest.

## Supporting information


Table S1.

## Data Availability

The data and code used in this study are available on the Dryad Digital Repository (https://doi.org/10.5061/dryad.59zw3r2h6). Water temperature data are available from Gewässerkundlicher Dienst Bayern (2024, https://www.gkd.bayern.de/de/fluesse/wassertemperatur/kelheim/kelheim‐10053009/messwerte/tabelle). Discharge data are available from Hochwassernachrichten Dienst Bayern (2024, https://www.hnd.bayern.de/pegel/donau_bis_kelheim/kelheim‐10053009/tabelle?methode=abfluss&).
