# Supplementary material for: Characterization of the reproductive strategy of invasive Round Goby (Neogobius melanostomus) in the Upper Danube River
Source: Ecol Evol. 2024 Oct 1;14(10):e70349. doi: 10.1002/ece3.70349 (PMC11445448; doi:10.1002/ece3.70349)
Supplement: Supplementary file 1 — Table S1. [file ECE3-14-e70349-s001.docx]

# Appendix

S 1: Information on fish assemblage in the Upper Danube. Status (invasive, non-native, native), number (n), minimum length (Min.length), maximum length (Max. length), median, mean and proportion of total catch (% total catch) are given for all fish caught during electrofishing campaigns in order of decreasing abundance.

| Common name | Scientific name | Status | n | Min. length (cm) | Max.  length  (cm) | Median  (cm) | Mean  (cm) | % total catch |
| --- | --- | --- | --- | --- | --- | --- | --- | --- |
| Round Goby | *Neogobius melanostomus* | invasive | 1428 | 2.1 | 15.6 | 6.0 | 6.3 | 44.8 |
| European Perch | *Perca fluviatilis* | native | 850 | 3.8 | 26.2 | 8.3 | 8.8 | 26.6 |
| Tubenose goby | *Proterorhinus marmoratus* | invasive | 292 | 1.6 | 11.2 | 6.0 | 6.3 | 9.2 |
| Roach | *Rutilus rutilus* | native | 209 | 1.0 | 17.7 | 7.5 | 7.7 | 6.5 |
| Chub | *Squalius cephalus* | native | 118 | 2.0 | 26.5 | 8.0 | 8.2 | 3.7 |
| Bleak | *Alburnus alburnus* | native | 61 | 0.9 | 16.0 | 3.5 | 4.4 | 1.9 |
| Pumpkinseed | *Lepomis gibbosus* | invasive | 35 | 4.3 | 12.5 | 9.0 | 9.2 | 1.1 |
| Eel | *Anguilla anguilla* | non-native | 29 | 4.1 | 70.0 | 38.0 | 35.7 | 0.9 |
| Dace | *Leuciscus leuciscus* | native | 28 | 5.2 | 6.8 | 6.9 | 6.8 | 0.9 |
| Asp | *Aspius aspius* | native | 23 | 5.5 | 12.0 | 7.3 | 7.5 | 0.7 |
| Bighead goby | *Ponticola kessleri* | invasive | 20 | 3.0 | 13.0 | 6.1 | 6.3 | 0.6 |
| Ide | *Leuciscus idus* | native | 17 | 5.0 | 14.5 | 7.4 | 8.0 | 0.5 |
| Barbel | *Barbus barbus* | native | 12 | 5.0 | 16.5 | 5.9 | 7.9 | 0.4 |
| Pikeperch | *Sander lucioperca* | native | 9 | 4.8 | 18.1 | 12.0 | 11.5 | 0.3 |
| Catfish | *Silurus glanis* | native | 9 | 8.1 | 55.0 | 13.5 | 20.5 | 0.3 |
| Common carp | *Cyprinus carpio* | native | 8 | 10.4 | 17.6 | 14.5 | 14.3 | 0.3 |
| Prussian carp | *Carassius gibelio* | native | 7 | 8.0 | 13.3 | 11.2 | 10.9 | 0.2 |
| Three-spined Stickleback | *Gasterosteus aculeatus* | native | 7 | 1.5 | 2.4 | 2.0 | 1.9 | 0.2 |
| Burbot | *Lota lota* | native | 4 | 17.0 | 32.0 | 23.6 | 24.1 | 0.1 |
| Tench | *Tinca tinca* | native | 4 | 3.0 | 5.5 | 5.3 | 4.8 | 0.1 |
| Spirlin | *Alburnoides bipunctatus* | native | 3 | 4.4 | 6.5 | 6.0 | 5.6 | 0.09 |
| Racer goby | *Babka gymnotrachelus* | invasive | 3 | 5.4 | 9.0 | 4.9 | 5.3 | 0.09 |
| Nase | *Chondrostoma nasus* | native | 3 | 4.6 | 8.8 | 7.0 | 6.8 | 0.09 |
| Gudgeon | *Gobio gobio* | native | 3 | 12.4 | 15.6 | 14.9 | 14.3 | 0.09 |
| Eurasian ruffe | *Gymnocephalus cernua* | native | 2 | 9.6 | 13.0 | 11.3 | 11.3 | 0.06 |
| Rudd | *Scardinius erythrophthalmus* | native | 2 | 5.4 | 10.5 | 8.0 | 8.0 | 0.06 |
| Crucian carp | *Carassius carassius* | native | 1 | 8.2 | 8.2 | 8.2 | 8.2 | 0.03 |
| Northern Pike | *Esox lucius* | native | 1 | 43 | 43 | 43 | 43 | 0.03 |
| Brook lamprey | *Lampetra planeri* | native | 1 | 20 | 20 | 20 | 20 | 0.03 |
| Topmouth gudgeon | *Pseudorasbora parva* | invasive | 1 | 8.9 | 8.9 | 8.9 | 9.0 | 0.03 |
| European bitterling | *Rhodeus amarus* | native | 1 | 4.0 | 4.0 | 4.0 | 4.0 | 0.03 |
